# Key Features of Smart Medication Adherence Products: Updated Scoping Review

**DOI:** 10.2196/50990

**Published:** 2023-12-19

**Authors:** Sadaf Faisal, Devine Samoth, Yusra Aslam, Hawa Patel, SooMin Park, Bincy Baby, Tejal Patel

**Affiliations:** 1 School of Pharmacy University of Waterloo Kitchener, ON Canada; 2 Schlegel – University of Waterloo Research Institute of Aging Waterloo, ON Canada; 3 Centre for Family Medicine Family Health Team Kitchener, ON Canada

**Keywords:** technology, medication, aging, adherence, smart medication, digital technology, self-management, older adult, mobile health, mHealth, apps, digital health, geriatrics, older adults, mHealth app, application, management, scoping review, medication adherence, consumer, use, mobile phone

## Abstract

**Background:**

Older adults often face challenges in self-managing their medication owing to physical and cognitive limitations, complex medication regimens, and packaging of medications. Emerging *smart* medication dispensing and adherence products (SMAPs) offer the options of automated dispensing, tracking medication intake in real time, and reminders and notifications. A 2021 review identified 51 SMAPs owing to the rapid influx of digital technology; an update to this review is required.

**Objective:**

This review aims to identify new products and summarize and compare the key features of SMAPs.

**Methods:**

Gray and published literature and videos were searched using Google, YouTube, PubMed, Embase, and Scopus. The first 10 pages of Google and the first 100 results of YouTube were screened using 4 and 5 keyword searches, respectively. SMAPs were included if they were able to store and allowed for the dispensation of medications, tracked real-time medication intake data, and could automatically analyze data. Products were excluded if they were stand-alone software applications, not marketed in English, not for in-home use, or only used in clinical trials. In total, 5 researchers independently screened and extracted the data.

**Results:**

This review identified 114 SMAPs, including 80 (70.2%) marketed and 34 (29.8%) prototypes, grouped into 15 types. Among the marketed products, 68% (54/80) were available for consumer purchase. Of these products, 26% (14/54) were available worldwide and 78% (42/54) were available in North America. There was variability in the hardware, software, data collection and management features, and cost of the products. Examples of hardware features include battery life, medication storage capacity, availability of types and number of alarms, locking features, and additional technology required for use of the product, whereas software features included reminder and notification capabilities and availability of manufacturer support. Data capture methods included the availability of sensors to record the use of the product and data-syncing capabilities with cloud storage with short-range communications. Data were accessible to users via mobile apps or web-based portals. Some SMAPs provided data security assurance with secure log-ins (use of personal identification numbers or facial recognition), whereas other SMAPs provided data through registered email addresses. Although some SMAPs were available at set prices or free of cost to end users, the cost of other products varied based on availability, shipping fees, and subscription fees.

**Conclusions:**

An expanding market for SMAPs with features specific to at-home patient use is emerging. Health care professionals can use these features to select and suggest products that meet their patients’ unique requirements.

## Introduction

### Background

As of July 2022, Canada’s older adults aged ≥65 years comprised 18.8% of the total population [1]. The number of centenarians per 100,000 persons has also increased from 20.5 (2001) to 34.6 [1]. This can be attributed to the baby boomers, the largest generation in Canadian history, reaching this age group between 2031 and 2036, as well as the gradual rise in life expectancy, which is projected to continue to increase [2]. Older adults are frequently diagnosed with multiple comorbidities, including cardiovascular diseases, diabetes, arthritis, and respiratory disorders [3]. In Canada, 1 out of 3 older adults aged 65 years is reported to have at least 2 chronic medical conditions [4].

Medications are the mainstay of treatment to manage chronic medical conditions. Despite the evidence that shows that medication adherence is vital for managing chronic diseases, medication nonadherence is common among older adults. This can be attributed to a variety of factors, such as cognitive impairment, polypharmacy, multimorbidity, drug-related adverse effects, and storage or formulation issues with medications [5]. Furthermore, older adults often face certain cognitive, physical, or both types of limitations that make daily medication management a difficult and demanding task. Inadequate medication adherence is associated with worsened clinical outcomes, decreased quality of life, and frequent hospitalizations. A retrospective cohort study addressing geriatric nonadherence among patients with heart failure reported that, with every 10% increase in medication adherence, there was a consequent 11% decrease in emergency room visits, 6% decrease in hospital admissions, and 9% reduction in mortality [6]. Similarly, medication nonadherence has been shown to have a negative impact on health care system costs. A literature review conducted by Cutler et al [7] estimated that 10% of hospitalizations occurred because of adherence issues. A nonadherent patient on average requires 3 more medical visits per year, which is typically a US $2000 increase in yearly health care costs [7]. Thus, medication nonadherence can be considered a critical clinical and economic problem.

Numerous interventions have been identified and used to support medication management in older adults [8]. One such intervention is the use of smart technology-based adherence products, otherwise called smart medication dispensing and adherence products (SMAPs) [9,10]. SMAPs contain sensors and processors that allow them to track real-time medication intake and record medication events such as the date and time of medication administration [11]. These products capture medication intake data through a human-initiated action such as opening a pill bottle, puncturing a blister pack, or pressing a button on an automated dispensing device. SMAPs allow for the communication of adherence information to patients, caregivers, and health care providers through the remote upload of data via various means of connectivity, including Wi-Fi, Bluetooth, long-term evolution, and near-field communication [11]. In 2020, our research team systematically searched for and identified 51 products, of which 38 were available for patients to purchase for in-home use [11]. In another recent review, 79 different technologies were found to be available for medication adherence, such as electronic pill boxes, pill bottles, blister packages, and various other products that can track medication intake in real time [12]. Although the rapid development of these technologies is intended to address medication nonadherence, it is not clear whether all of these products positively affect medication taking. For example, the results of a previous scoping review examining the usability, acceptability, and functionality of smart oral multidose dispensing systems indicated that the impact of these systems on medication adherence was inconsistently defined, measured, and reported in the studies [13]. In addition, although acceptability and usability were reported by the studies included in the scoping review, the details of why a product was usable or acceptable were not specified. The use of these SMAPs may be driven by user experience with the features offered by specific products. For example, a usability study conducted by Patel et al [14] reported that the usability of different electronic medication adherence products was highly variable. The variability in the usability of these products may be dependent on their features as well as the medication management capacity of the individuals using these products. Previous qualitative research indicates that older adults, caregivers, and health care providers identified simplicity, availability and usability of alarms, portability, restricted access to medications, and storage capacity as some of the product features that may drive their decision-making regarding the use of a product to support medication management [15]. The same study also highlighted user factors that may drive the appropriate use of these technologies, including sentiment, privacy, user frustration, affordability, physical and cognitive capacity, and technology literacy and learnability. Medication adherence technologies that are not usable by patients may negatively affect medication adherence (ie, worsen medication nonadherence rather than improve it). However, the features of different medication adherence technologies that may drive the usability of these products have not been previously outlined.

### Objectives

Given the rapid development of such technology and how features of different products may need to be considered by clinicians when recommending smart medication adherence technologies, we sought to update a previous review of the features of new SMAPs available for in-home use [16,17]. Information on the available features of SMAPs is especially important to clinicians addressing medication nonadherence among their patients and can assist with the selection of the best-suited product. Therefore, the primary objective of this review was to identify SMAPs available worldwide and describe and compare their features to assist clinicians in recommending products that suit their patients’ needs, expectations, and capacity to improve adherence.

## Methods

A systematic approach was used to search for and maximize the number of products identified. The search encompassed both published and gray literature to identify the maximum number of SMAPs available.

### Search Strategy for Published Literature

Published literature was searched using 3 databases: PubMed (MEDLINE), Ovid Embase, and Scopus. The search strategy was developed by consulting a librarian. Keywords and Medical Subject Heading terms, including “medication,” “adherence,” “smart,” and “dispensing,” were used to search the databases. The Boolean operators AND/OR were used to combine the search terms. Multimedia Appendix 1 provides the detailed search strategy for the databases. The searches were limited to between January 2019 and August 2022 to identify the scholarly articles published since our last review. One researcher (SF) conducted the searches. The final search was conducted on September 29, 2022. All citations were imported to Mendeley Desktop (version 1.19.4; Elsevier Ltd), and duplicates were removed. The titles and abstracts of the search results were reviewed by 1 researcher (SF) to identify all potential SMAPs. A full-text review of potentially relevant citations was completed by a single researcher (SF) to extract the product information. We used the PRISMA (Preferred Reporting Items for Systematic Reviews and Meta-Analyses) guidelines for reporting the studies.

### Search Strategy for Gray Literature

Gray literature was searched using the Google and YouTube search engines with the keywords “smart medication dispensers,” “smart medication device,” “smart dispensing delivery,” “smart blister pack,” and “smart medication vial.” Multimedia Appendix 2 provides the detailed search strategy for YouTube and Google. In total, 2 researchers (HP and SP) conducted the Google and YouTube searches independently. The researchers searched the first 10 pages of Google and the first 100 YouTube videos using each search strategy to identify products that met the inclusion criteria. In total, 2 other researchers (YA and DS) reviewed the included products and extracted the product details.

### Inclusion and Exclusion Criteria

Products were included in this review if they (1) were smart, defined as “products that are embedded with processors or sensors that allow data to be exchanged between the products and its environment, manufacturer, user and other product systems automatically via various means of connectivity” [18]; (2) had a mechanism to either dispense or organize medications; and (3) were able to track real-time medication intake via mobile apps or web-based portals.

Products were excluded if (1) they were not available for in-home patient use, (2) the product information was not available in English, (3) they were stand-alone mobile apps, or (4) were used only in clinical trials.

### Data Extraction

The assessment criteria developed by Mason et al [12], in addition to features assessed in our previous review, were used to extract data related to the SMAPs. The assessment criteria developed by Mason et al [12] included technology hardware and software features, development information, data collection and management, feasibility and implementation, and acceptability and usability. Data related to the products and their features were independently extracted by 4 researchers (SF, BB, HP, and SP) and reviewed and discussed within the team. The following features were extracted: (1) type of products based on their design; (2) developer information, including region or country of product availability, development stage, regulatory approval status, and commercial availability; (3) technological features, including hardware (size, battery life, product storage capacity, alarms, secure medication storage, portability, and additional technology needed to use the product) and software (reminders, notifications, available customer support for users, and ability to integrate with other clinical systems) features; (4) data collection and management; and (5) other features, including product adoption or social engagement via the product.

## Results

### Overview

**Figure 1:**
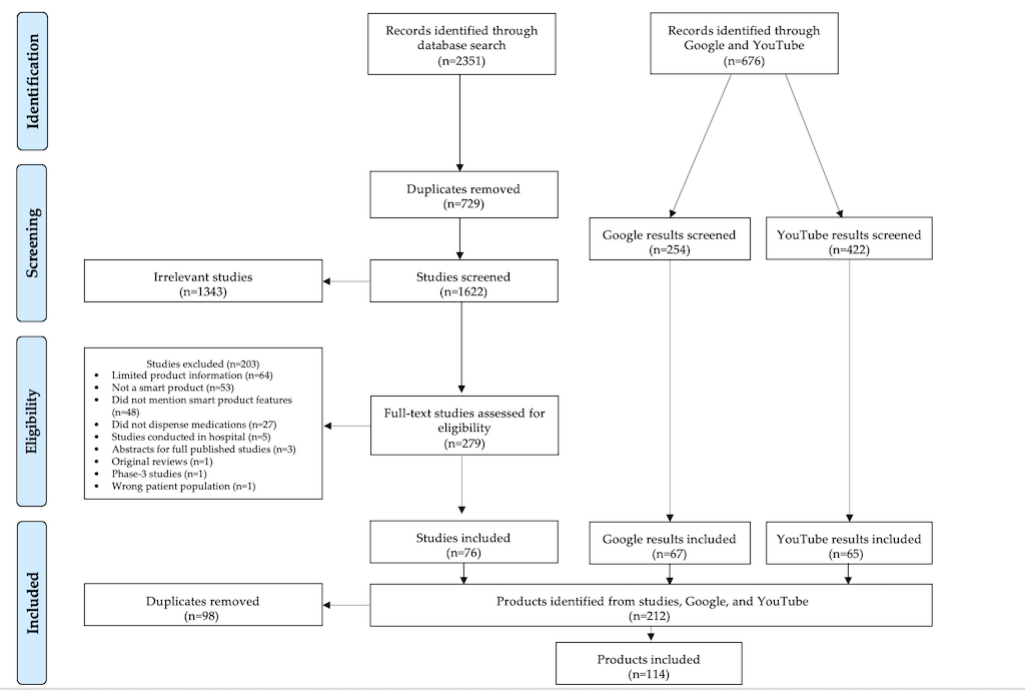
PRISMA (Preferred Reporting Items for Systematic Reviews and Meta-Analyses) flow diagram.

The published literature search identified 2351 studies from the databases—PubMed (n=1169, 49.72%), Embase (n=722, 30.71%), and Scopus (n=460, 19.57%)—and an additional 676 records were identified from Google (n=254, 37.6%) and YouTube (n=422, 62.4%). Figure 1 shows the PRISMA flow diagram.

From these searches, we identified a total of 212 products, including 80 (37.7%) products identified from 76 research studies, 67 (31.6%) from Google, and 65 (30.7%) from YouTube. After removing 98/212 (46.2%) duplicates, the final review, the final review included 114 products. The products were grouped into 15 categories. The definitions of the different types of products are outlined in Textbox 1.

The review included 114 products, of which 34 (29.8%) were prototypes and 80 (70.2%) were marketed. Prototypes were defined as preliminary models of a product that were not fully developed, and marketed products were defined as fully developed products that were available for purchase. Tables 1-5 provide the lists of the marketed and prototype SMAPs that were included in this review.

**Definitions.**

**Product type**

- Automated dispenser: medication dispensers that provide access to a controlled number of medications at specific time intervals via human-initiated actions and record dispensation or retrieval of medications
- Blister pack: plastic packaging that holds medications in individual pockets or compartments and is sealed using adhesive-coated paper or aluminum that incorporates electronic circuitry or conductive wiring
- Blister pack add-on sensor: sensors that can be attached to a traditional blister pack and record the puncturing of different blisters or compartments in the pack
- Blister pack holder: a box or case that allows for the storage of blister packs or blister cards and records the time at which the box is opened
- Inhaler: electronic inhaler with built-in sensors that automatically records use
- Inhaler add-on sensor: a sensor that can be attached to an inhaler device and track the date and time of inhaler use; some inhalers have the ability to track other respiratory parameters such as inhalation duration or flow rate
- Inhaler holder: a stand or holder that is designed for the purpose of holding or storing 1 or more inhalers and automatically records data when the inhaler is used
- Injectors: electronic injector with built-in sensors that automatically records use
- Injector holder: a stand or holder that is designed for the purpose of holding or storing 1 or more injectables and automatically records data when the injector is used
- Medication tray: multicompartment medication organizers for storing medications, with built-in sensors that automatically record the date and time of opening the compartment
- Medication tray holder: a box or case that allows for the storage of medication vials or a medication tray and records the time at which the box is opened
- Pill bottle: a vial that can store medication and automatically records the date and time when the lid is opened
- Pill bottle holder: a stand or holder that is designed for the purpose of holding or storing 1 or more pill bottles and automatically records the date and time when a pill bottle is removed from the holder
- Pill box: a box that can contain medications in their original packaging and that have not been repackaged into pouches, blisters, or compartments and automatically records the date and time when it is opened
- Vial caps: sensors on the vial cap that record the date and time when the vial is opened

**Product features**

- Alarm: use of visual or auditory signals produced by the device to alert the user to the time of dosing
- Reminder: a written or audio message that reminds users to perform an activity, such as medication taking
- Notification: an alert (typically a pop-up or other message) generated by an application to notify the user of a new message, update, social media post, missed dose, or wrong dose
- Accessibility: design of products, devices, services, or environments for people who experience disabilities
- Single-medication storage: a product that can only store 1 type of medication
- Multimedication storage: a product that can store >1 type of medication
- Ability to integrate with clinical platforms: for example, pharmacy software, electronic medication administration records, and hospital records
- IT support: for example, phone number, email, web-based forms, and web-based chats for customer inquiry about any technical difficulties with the product

**Table 1:** Marketed and prototype smart medication adherence products—automated dispensers.

Product type and product name				Manufacturer or supplier		Products, n (%)
**Marketed (n=80)**						
	**Automated dispenser**					24 (30)
		EMMA [11]	EMMA Health Technologies			
		e-Pill MedSmart PLUS [19]	e-Pill			
		Evondos [20]	Evondos			
		Hero pill dispenser [21-26]	Hero Health			
		Home8 Medication Dispensing System [27-29]	Visiotech			
		Karie [30-32]	AceAge Inc; Karie Health			
		LiveFine [33,34]	LiveFine			
		Livi [35,36]	PharmRight Corp			
		MedaCube [37,38]	PharmAdva			
		Medido [39]	Innospense BV			
		MedReady [40,41]	MedReady, Inc; TabTimer			
		Mymemo [42]	Studio Volpi			
		Philips Medication Dispenser [17,22,43,44]	Philips Lifeline			
		Pill Connect [45-50]	eLucid mHealth Ltd			
		Pivotell Advance GSM [51]	Pivotell			
		Pria [52,53]	Black & Decker			
		RxPense Care [54]	Medipense			
		DoseControl Smart Automatic Pill Dispenser—Model 2021 [55-57]	MedControl			
		Smart medication dispenser [58,59]	As Directed TLC^a^			
		Smart Pillbox medicine management system [60]	BlueStar SeniorTech			
		Spencer [61-67]	Custom Health Inc			
		TabSafe [68]	Medication Solutions			
		The Kindo [69]	KIN			
		Voice Pro (e-Pill MedSmart PLUS) [19,70]	e-Pill			
**Prototypes (n=34)**						
	**Automated dispenser**					5 (15)
		e-pill smart medication dispenser [71]	CybernetX			
		ReX [13,72]	Dosentrx Ltd			
		Smart medicine dispenser [73]	—^b^			
		Smart pill dispenser [74]	—			
		Smart pill expert system [75]	Unknown			

^a^TLC: Total Linked Care.

^b^Not available.

**Table 2:** Marketed and prototype smart medication adherence products—blister packs.

Product type and product name				Manufacturer or supplier		Products, n (%)
**Marketed (n=80)**						
	**Blister pack**					6 (8)
		Popit [76]	Popit			
		Electronic blister pack (Med-ic) [77]	IMC^a^			
		SMART Blister Pack [78]	Wellness Pharmacy			
		Smart Clinical Support Package [79]	ECCT B.V.			
		Smart Polypharmacy Card [80]	ECCT B.V.			
		Time4Med [81]	Adherence Innovations			
	**Blister pack holder**					2 (3)
		Aavia smart birth control pill case [82]	Aavia			
		Sensemedic Blister Dispenser [83,84]	Evalan			
	**Blister pack sensor**					1 (1)
		CueSticker [85,86]	CuePath Innovation			
**Prototypes (n=34)**						
	**Blister pack**					4 (12)
		CpaX [87,88]	Jones Healthcare Group			
		Electronic medication blister [89]	DSM TCG B.V.			
		SmartBlister [90]	ECCT B.V.			
		Smart blister [91]	Unknown			

^a^IMC: Information Mediary Corp.

**Table 3:** Marketed and prototype smart medication adherence products—eye drops, glasses, inhalers, and injectables.

Product type and product name				Manufacturer or supplier		Products, n (%)	
**Marketed (n=80)**							
	**Inhaler**						12 (15)
		Digihaler (ProAir, AirDuo, and ArmonAir) [92-94]	Teva Pharmaceutical Industries				
		Enerzair Breezhaler [94,95]	Novartis Europharm Limited				
		FindAir ONE [96]	FindAir				
		Hailie sensor [92,93,97-99]	Adherium				
		Propeller [92,93]	Propeller Health (ResMed)				
		Respiro device [92,94,100,101]	Amiko				
		SmartDisk [92,94]	Nexus6 Ltd				
		SmartMat [92,102]	Nexus6 Ltd				
		SmartTouch [92]	Nexus6 Ltd				
		SmartTrack [92]	Nexus6 Ltd				
		SmartTurbo [92,94]	Nexus6 Ltd				
		Turbu+ V2.1 [103]	AstraZeneca UK Ltd				
	**Inhaler add-on sensor**						2 (3)
		FindAir (pMDI^a^, Turbuhaler, Ellipta, and Easyhaler) [96]	FindAir				
		HeroTracker [104]	Aptar Pharma				
	**Injector**						4 (5)
		BETACONNECT autoinjector [105]	Bayer				
		Easypod [106-110]	Merck Serono International S.A.				
		InPen [111,112]	Medtronic				
		RebiSmart [105]	Merck KGaA				
	**Injector holder**						1 (1)
		SmartSyringe [113]	ECCT B.V.				
**Prototypes (n=34)**							
	**Eye drop device**						2 (6)
		KaliDrop device [114]	Kali Care				
		Smart electronic eye drop bottle [115]	—^b^				
	**Glasses**						1 (3)
		MedGlasses (smart glasses-based pill recognition system) [116]	—				
	**Inhaler**						2 (6)
		SmartTrack device [117]	Shanghai Sonmol Internet Technology Co, Ltd				
		VHC^c^ [118]	InspiRx, Inc				
	**Inhaler add-on sensor**						2 (6)
		BreatheMate [119,120]	AstraZeneca				
		FindAir (Diskus; capsules) [96]	FindAir				

^a^pMDI: pressurized metered dose inhaler.

^b^Not available.

^c^VHC: valve-holding chamber.

**Table 4:** Marketed and prototype smart medication adherence products—medication trays and pill boxes.

Product type and product name				Manufacturer or supplier		Products, n (%)	
**Marketed (n=80)**							
	**Medication tray**						5 (6)
		Ellie [121]	EllieGrid				
		MedMinder Maya [122,123]	MedMinder				
		MedMinder Jon [11,122,124-126]	MedMinder				
		MED-TIMER [127]	Arthritis Supplies				
		SimpleMed+ [128,129]	Vaica Medical				
	**Medication tray holder**						2 (3)
		Sensemedic Pill Dispenser [83,84]	Evalan				
		Wisepill RT3000 [130]	Wisepill Technologies				
	**Pill box**						9 (11)
		CleverCell [131]	Compliance Meds Technologies				
		CYCO [132]	Qualife				
		evriMED1000 [133]	Wisepill Technologies				
		iLidRx [134,135]	iRxReminder LLC				
		Pillgo [136]	Pillgo				
		Pletaal Assist System [137]	Otsuka Pharmaceutical				
		Smart narrowband-IoT^a^ Pillsure Pocket [138,139]	1NCE				
		Wisepill 2G (model RT2000) dispenser [13,130,140-148]	Wisepill Technologies				
		Wisepill 4G LTE^b^ (model RT2000) medication dispenser [130]	Wisepill Technologies				
**Prototypes (n=34)**							
	**Medication tray**						4 (12)
		EDossette [13,149]	McMaster University				
		mHealth^c^ system [150]	MedMinder				
		SMSS^d^ [13,151]	—^e^				
		Smart pill box [152]	—				
	**Pill box**						5 (15)
		iMedBox [153]	—				
		IoT device [154]	—				
		KaliJAR [155]	Kali Care				
		OnDosis [156]	—				
		Smart pill box [157]	—				

^a^IoT: Internet of Things.

^b^LTE: long-term evolution.

^c^mHealth: mobile health.

^d^SMSS: smartphone-based medication self-management system.

^e^Not available.

**Table 5:** Marketed and prototype smart medication adherence products—pill bottles and vial caps.

Product type and product name				Manufacturer or supplier		Products, n (%)
**Marketed (n=80)**						
	**Pill bottle**					7 (9)
		Aidia [158-169]	AdhereTech			
		CleverCap Lite [170]	Compliance Meds Technologies			
		SmartBottle [171]	ECCT B.V.			
		eCAP [172]	IMC^a^			
		Nomi Bottle [173,174]	SMRxT Inc			
		Pilleve [175,176]	Pilleve Inc			
		Pillsy Smart Cap [177,178]	Pillsy Inc			
	**Pill bottle holder**					1 (1)
		Sensemedic Pill Bottle Dispenser [84]	Evalan			
	**Vial cap**					4 (5)
		SmartCap [171]	ECCT B.V.			
		CleverCap Pro [131]	Compliance Meds Technologies			
		Smart Med Reminder System [179]	Concordance Health Solutions			
		SmartVial [180]	ECCT B.V.			
**Prototypes (n=34)**						
	**Pill bottle**					5 (15)
		Electronic pill bottles [181,182]	Digital Media Technologies and AdhereTech			
		EVE smart bottle [183]	—^b^			
		MotionDx [184]	—			
		Smart pill bottle [185]	—			
		Smart pill bottle [186]	Pfizer			
	**Pill bottle holder**					2 (6)
		NVOLVE [187]	NVOLVE			
		SMRT^c^ bottle [188]	Amcor and Confrérie Clinique			
	**Vial cap**					2 (6)
		BETR-Cap [189]	Pacific Life Technologies			
		Pill-safe digital health system [190]	ModoScript			

^a^IMC: Information Mediary Corp.

^b^Not available.

^c^SMRT: separate, monitor, release, and track.

### Consumer Availability

**Figure 2:**
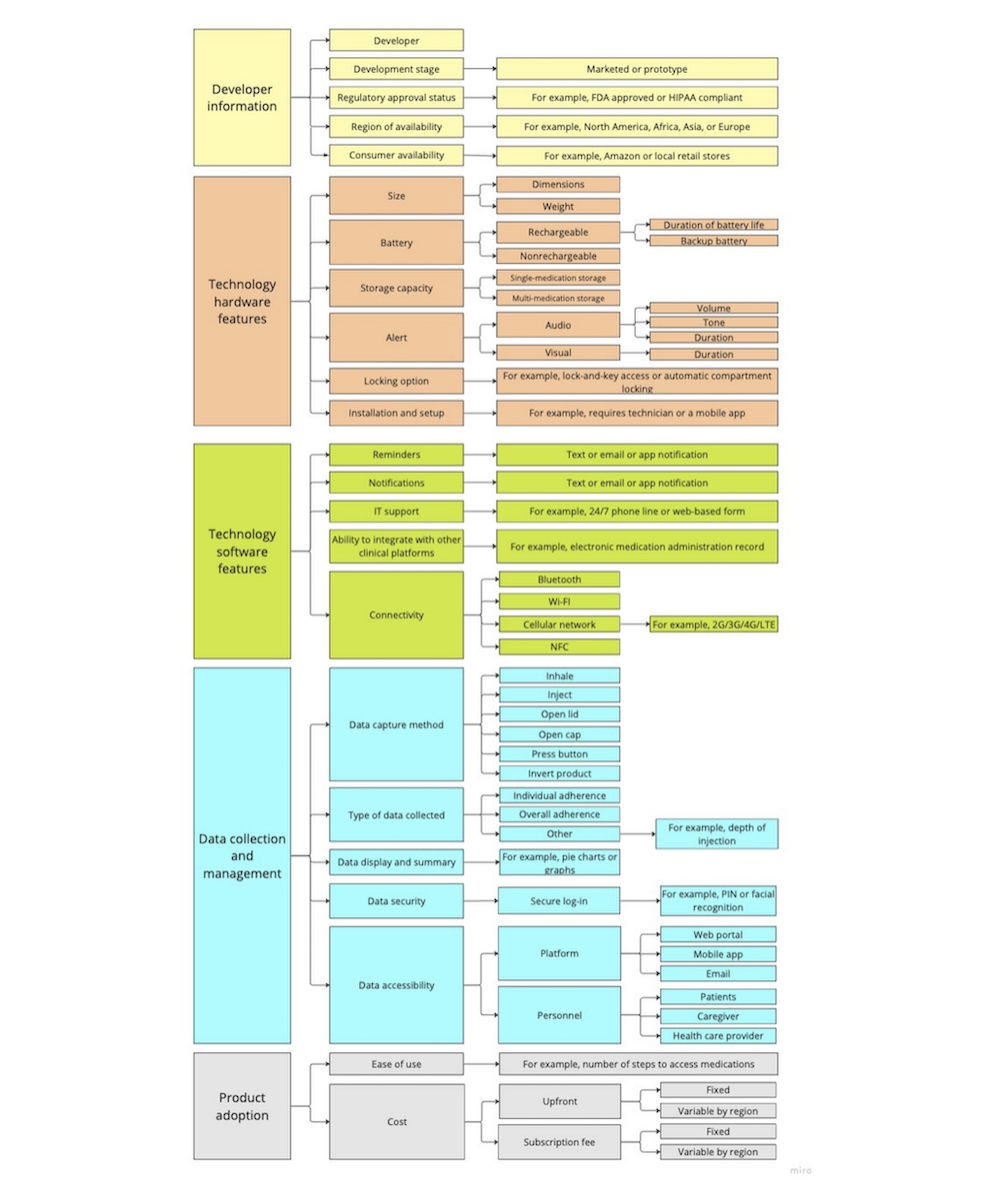
Smart medication adherence product features. eMAR: electronic medication administration record; FDA: Food and Drug Administration; HIPAA: Health Insurance Portability and Accountability Act; LTE: long-term evolution; NFC: near-field communication; PIN: personal identification number.

Among the 80 marketed products (Table 6), 54 (68%) were available for consumer purchase, whereas the remaining 26 (33%) were available to patients through partner organizations or for clinical research purposes only (Table 6 and Figure 2). Of the products available for consumer purchase, 26% (14/54) were available worldwide, 78% (42/54) were available in North America, 30% (16/54) were available in South America, 44% (24/54) were available in Europe, 30% (16/54) were available in Australia, 35% (19/54) were available in Africa, and 44% (24/54) were available in Asia.

**Table 6:** Marketed smart medication adherence product features.

Product feature				Products, n (%)
**Consumer availability (n=54)**				
	Africa		19 (35)	
	Asia		24 (44)	
	Australia		16 (30)	
	Europe		24 (44)	
	North America		42 (78)	
	South America		16 (30)	
	Worldwide		14 (26)	
**Hardware features (n=80)**				
	**Battery life**			
		≤1 y	33 (41)	
		>1 y	9 (11)	
		Not reported	38 (48)	
	**Storage capacity**			
		Single-medication storage	21 (26)	
		Multimedication storage	57 (71)	
		Not reported	2 (3)	
	**Alarm**			
		None	4 (5)	
		Audio only	10 (13)	
		Visual only	5 (6)	
		Both audio and visual	44 (55)	
		Not reported	31 (39)	
	**Locking feature**			
		Available	19 (24)	
		Not available	22 (28)	
		Not reported	39 (49)	
	**Installation and** **setup**			
		Additional app required	61 (76)	
		No additional app required	13 (16)	
		Not reported	6 (8)	
**Software features (n=80)**				
	Reminders		60 (75)	
	Notifications		50 (63)	
	Available IT support		35 (44)	
	Ability to integrate with other clinical platforms		5 (6)	
	Ability to capture other data or metrics		15 (19)	
	**Data accessibility**			
		Patient only	8 (10)	
		Patient and CPs^a^ only	22 (28)	
		Patient and HCPs^b^ only	12 (15)	
		Patient, CPs, and HCPs	28 (35)	
	**Data security**			
		Secure log-in	26 (33)	
		Not reported	54 (68)	
	**Connectivity**			
		Built-in SIM	8 (10)	
		Cellular data, Wi-Fi, or Ethernet required	72 (90)	
		Bluetooth	11 (14)	
		NFC^c^	8 (10)	

^a^CP: care provider (or caregiver).

^b^HCP: health care provider.

^c^NFC: near-field communication.

### Regulatory Approval

Regulatory approval (Table 6) refers to any authorization by the government or health authorities required to market a product in a given country. Examples of the types of regulations considered include the US Food and Drug Administration, Australian Quality Care Pharmacy Program, and Health Insurance Portability and Accountability Act compliance. There were 18.4% (21/114) of identified marketed products that had one or more of the described regulatory approvals, whereas 81.6% (93/114) of the marketed products’ regulatory approval status was not stated or indeterminate.

### Hardware Features

#### Battery Life

The hardware features are outlined in Tables 6 and 7 and Figure 2. Battery life is defined as the duration for which the product can be used after the battery is fully charged. Our review found that some products used single-use batteries that could not be recharged. Some products (4/114, 3.5%) were equipped with a battery level display to inform users of low battery before depletion, which allowed for a timely replacement (eg, Pivotell Advance GSM) [51]. However, some products (29/114, 25.4%) did not have this feature, which may cause them to stop working before users become aware that the battery needs replacement. For products using rechargeable batteries, a full charge could sustain function for up to 1 week, as in the case of Ellie, or up to 3 years (eg, Pill Connect) [45-50,121]. Other products may require an uninterrupted connection to a power outlet to function, which highlights concerns in case of a power outage or lack of access to power during travel (eg, Hero pill dispenser) [21-26]. However, some products may be equipped with a backup power supply to maintain their function for an additional 4 (eg, Pria) to 48 hours (eg, MedReady) in case of a power outage [40,41,52,53].

**Table 7:** Prototype smart medication adherence product features (N=34).

Product feature					Products, n (%)
**Hardware features**					
	**Storage capacity**				
		Single-medication storage	5 (15)		
		Multimedication storage	19 (56)		
		Not reported	10 (29)		
	**Alarm**				
		None	1 (3)		
		Audio only	2 (6)		
		Visual only	0 (0)		
		Both audio and visual	10 (29)		
		Not reported	21 (62)		
	**Locking feature**				
		Available	5 (15)		
		Not available	3 (9)		
		Not reported	26 (76)		
**Software features**					
	Reminders			34 (100)	
	Notifications			12 (35)	
	**Data accessibility**				
		Patient only	1 (3)		
		Patient and CPs^a^ only	5 (15)		
		Patient and HCPs^b^ only	6 (18)		
		Patient, CPs, and HCPs	8 (24)		
		Not reported	14 (41)		

^a^CP: care provider (or caregiver).

^b^HCP: health care provider.

#### Medication Storage Capacity

Single-medication storage products are designed to only store one type of medication (eg, pill bottles), as opposed to their counterpart, multimedication storage, which can store ≥2 types of medications (eg, automated dispensers). More than two-thirds of the currently marketed SMAPs (62/80, 78%) are multidose, 15% (9/62) hold a single type of medication whereas the remaining 85% (53/62) support multiple (≥2 ) medications storage capabilities. The number of prescriptions per dose, doses per day, and duration of the supply cycle can differ across products. For instance, LiveFine and Home8 Medication Dispensing System can supply medication for 28 days, storing up to 6 doses per day, whereas Livi has the capability to store 15 medications for a duration of 90 days, enabling the dispensing of a maximum of 24 doses each day [27-29,33-36].

#### Alarms

A total of 68% (54/80) of the marketed SMAPs were equipped with an alarm feature that reminded users to take the medication when scheduled doses were due. Of these 54 SMAPs, 10 (19%) provided only audio alarms that included beeping or chiming, 5 (9%) were equipped with only visual alarms (eg, a flashing light), and 44 (81%) were reported to have both audio and visual alarms. Some products (4/54, 7%) allowed for recurrent alerts before the dose was due, at the time of the dose, and after the dose. These alerts could continue up to a specified amount of time or until the dose was taken. At each alert, certain products may require users to click a button to confirm the number of pills to be dispensed and initiate dispensation (eg, Livi), whereas others (2/54, 4%) gave the option to receive a dose early or a missed dose (eg, MedaCube) [35-38]. Other information may also be present on the screen interface of the SMAP during dosage events, such as medication name, medication dose, and quantity of pills left (eg, Livi) [35,36].

#### Locking Feature

Approximately one-quarter of the products (24/114, 21.1%) were equipped with a locking mechanism through either lock and key–protected access to medication storage compartments (eg, MedaCube, e-pill MedSmart PLUS, and LiveFine) or automatic locking of upcoming dose compartments, permitting access only to compartments for doses that were due (eg, MedMinder Jon, Pill Connect, Pilleve, and Pria) [11,21,33,34,37,38,45,50,52,53,70,121-126,175,176]. Some locking mechanisms did not permit access on a scheduled dose basis; however, they did offer a child-resistant feature (eg, eCAP and Spencer), whereas some products (23/114, 20.2%) did not have any locking features (eg, MedMinder Maya). SMAPs that come with a locking feature may prevent users from accessing the wrong pills at the wrong time and may be used to prevent unauthorized or accidental access to medications (eg, child safety or limiting access to medications with high misuse potential) [61-67,122,123,172].

#### Additional Technology Required to Use the Product

SMAPs track and record real-time adherence data on an external web- or cloud-based platform. In most cases, the data recorded are accessible via a dashboard; thus, it may require the installation of additional software apps on mobile devices such as smartphones or tablets or on a computer or logging in through a web-based portal to complete the setup. There were 16% (13/80) of products that did not require any additional software installations, and they reported adherence through other means such as SMS text messaging or email.

### Software Features

#### Reminders and Notifications

The software features are outlined in Tables 6 and 7 and Figure 2. Approximately 75% (60/80) of the marketed SMAPs were equipped with the functionality of sending reminders, and 62% (50/80) were equipped with notifications. Reminders were written or spoken messages that reminded individuals to take their medications. In contrast, notifications were alerts (typically a pop-up or other message) generated by an application to notify the user of a new message regarding events other than taking medication, such as missed dose alerts, wrong-dose alerts, or other suggestions to help improve medication adherence. Most products (59/114, 51.8%) included both alerts and reminders. In addition to notifying patients, some products (6/114, 5.3%) provided the option of notifying care partners in cases of a missed dose or double dose (eg, Pillsy Smart Cap) [177,178,191-193].

#### Available Manufacturer Support Related to the Product

IT support refers to the ability of users to directly contact customer services for technical support—this does not include noninteractive user guide pamphlets that are presumed to come with the SMAPs. IT support is offered by the manufacturers to their consumers through a 24/7 phone line, email, SMS text messaging, or web page chat or form. This review identified 44% (35/80) of products that came with IT support. Other accessibility services included certified health care providers available on standby who could provide internet-based advice and home office support when needed. For instance, the Smart Pill Box Medicine Management Systems provide access to licensed nurses, whereas the Karie Pharmacy Home Monitoring Program provides access to licensed pharmacists, and nurses are available as support for users [30-32,60].

### Data Collection and Management

#### Data Capture Method

The data collection and management features are outlined in Tables 6 and 7 and Figure 2. SMAPs automatically record the date and time of use in response to a human-initiated physical action. For example, data capture occurs when a user opens the lid of a medication tray, opens vial caps or pill bottles, presses a button, or inverts the product. Some other methods for data capture included actuation of the inhaler or injector, opening the medication box, punching a blister, or pulling the tab in a blister pack. Depending on the product type, sensors were designed to automatically record the use of the product as an indicator of the dosage being taken. To be able to synchronize the collected data with cloud storage, SMAPs without a built-in SIM require one or more of the following short-range communications: wireless near-field communication, which was available on 10% (8/80) of the SMAPs in this review; Bluetooth (11/80, 14%); and cellular data (eg, 2G, 3G, or 4G and long-term evolution), Wi-Fi, or Ethernet on 90% (72/80) of the SMAPs in this review. SMAPs with a built-in SIM, which was available in 10% (8/80) of the products in this review, may require an additional subscription fee; however, they do not require wireless short-range communication (eg, Mymemo or Philips Medication Dispenser) [17,22,42-44].

#### Adherence Data Captured by the Product

SMAPs vary in their method of recording the date and time of medication taking. SMAPs may be designed to report periodic (ie, weekly, monthly, and annual) medication adherence history. Individual or overall adherence may be reported as percentages or presented in graphical form (eg, calendar view and pie charts).

#### Other Data Captured by the Product

A total of 19% (15/80) of the products were also designed to record other data in addition to medication adherence. For instance, the FindAir inhaler was able to capture pollutant levels in the surrounding environment at the location of use [96]. Similarly, Propeller was able to capture the location of use, and BETACONNECT autoinjectors could capture the depth of the injection [92,93,105]. Some products had the ability to capture one or more types of biometric data, such as blood pressure, blood glucose, heart rate, respiratory rate, and weight (eg, Pillgo, Respiro device, and Smart Pillbox Medicine Management System) [60,92-94,100,101,136].

#### Data Security and Accessibility

The medication adherence reports were accessible to patients through mobile apps (eg, Pletaal Assist System, Popit, Propeller, and RebiSmart), web-based portals (eg, Pivotell Advance GSM), or both mobile apps and portals (eg, RxPense Care). In total, 32% (26/80) of the SMAPs provided data security assurance for mobile apps and web-based portal access via secure log-ins (ie, personal identification number or facial recognition). For products that did not have application installations or portals in the initial setup, medication adherence reports were sent to registered email addresses (eg, Wisepill) [11,51,54,76,92,93,105,130,137,140-148]. Currently, only 6% (5/80) of the SMAPs automatically integrate adherence data into other clinical platforms such as pharmacy software or electronic medical records. Adherence reports could be accessed only by the patient in 10% (8/80) of the SMAPs, whereas 28% (22/80) permitted access by the patient and their caregivers; 15% (12/80) permitted access by the patient and their health care provider or providers; and 35% (28/80) permitted access by the patient, caregiver or caregivers, and health care provider or providers.

### Cost

There was a diverse range of prices for the different SMAPs. With suitable health insurance coverage, some products (2/114, 1.8%) could be free of cost (eg, Aidia and Medido) to the end user, whereas others (4/114, 3.5%) were available at discounted prices (eg, InPen) [39,111,112,158-169]. Some products (2/114, 1.8%) were offered at no cost if specified conditions were met—for instance, SMART Blister Packs were available free of cost for Wellness Pharmacy patients [78]. In contrast, Pria, along with Voice Pro (e-pill MedSmart PLUS), cost up to US $300 [19,52,53,70]. The cost of a product may also vary by region owing to a variety of factors, such as availability in the region or shipping fees (eg, LiveFine) [33,34]. In some cases (8/114, 7%), providers required users to directly contact them for more purchase information (eg, Livi and Nomi Bottle) [35,36,173,174]. In addition to the upfront cost of the product, some products (8/114, 7%) required monthly or annual subscription fees. Among these products, some (2/114, 1.8%) offered a free trial period before the subscription started (eg, Time4Med and TabSafe) [68,81].

## Discussion

### Principal Findings

This scoping review provided a summary of emerging SMAPs, including both prototypes and marketed products. This review also provided detailed descriptions of their features, including product type, hardware features (battery life, storage capacity, alarms, and locking ability), software features (ie, reminders, notifications, IT support, and integration with other clinical platforms), data collection and management, and product cost. A comprehensive comparison of the product features can inform patients, their care partners, and clinicians as they assess the benefits and challenges of using such a product to support self-management of medications. For instance, a product with multimedication storage capacity and an audio alarm may be more suitable for patients with visual impairment who are self-managing a complex medication regimen with multiple medications. Similarly, the ability to limit access to medications using locking features may be essential for patients with cognitive impairments to limit overadherence or in homes where children may be cared for. Reminders and alarm functions are important features of SMAPs that can help improve medication adherence [194]. SMAPs with audio or visual alarms or reminders inform users when a scheduled dose is due, and for some products, the alarm will continue until the dose is taken (ie, MedReady) [40,41]. This feature can be valuable for forgetful older adults.

The availability of SMAPs with a variety of features provides end users with a range of products to choose from when deciding on a device to support medication self-management. However, for these products to be adopted by older adults, several factors need to be addressed. Product design and cost were identified as barriers to use by older adults in a qualitative study investigating the integration of a prototype smart blister pack among older adults with chronic diseases [11]. Product design affects usability, and it is vital to determine whether these products are usable. A previous study examining the usability of 21 electronic medication adherence products demonstrated that their usability varied widely [14]. Although SMAPs provide the ability to monitor medication taking in real time, if older adults are not able to appropriately use these products, it may worsen medication taking rather than improve adherence. An important feature identified in this review is the offer of additional IT support or 24/7 customer service. This factor can improve the usability of the product to accommodate a wider range of users, including those who lack confidence with technology.

Our review identified that these products ranged variably in terms of cost, from a few to a few hundred dollars, whereas others were available without cost to the end user. Still, financial consideration is important as many older adults live on limited income [12].

SMAPs record medication intake in real time and transmit these data to a mobile app or portal. These apps display and summarize the adherence data and allow for remote access, which provides not only the ability for patients to be aware of their medication taking but also the opportunity for caregivers and health care providers to conveniently access an overview of patients’ adherence history. Research has reported that health care providers value the availability of real-time medication-taking data and perceive that access to medication adherence data can help make clinical decisions in a timely manner, thus improving health-related outcomes for patients [13,15]. However, although SMAPs can track real-time data, they do not ensure that the patient ingested, inhaled, or injected the medication and only provide surrogate markers for adherence. Hence, there is room for discrepancy between the adherence data recorded and patients’ actual adherence. The ability of SMAPs to integrate with other clinical platforms such as pharmacy software, electronic medication administration record, or hospital records is also highly valued by stakeholders, especially pharmacists and physicians, as it allows them to access adherence data in a seamless manner and may reduce their workload [15].

In our previous review, published in 2021, we identified 51 SMAPs. This scoping review identified a total of 114 SMAPs. Over a period of 4 years, the number of SMAPs has doubled, providing users with more options and variability in the products they can use. The increase in the number of products underlines the importance of addressing the declining capacity of older adults to self-manage their medications, which has downstream effects on medication adherence, medication errors, clinical outcomes, and hospitalizations.

The major strength of this review is the use of both published and gray literature to identify the products as well as using a comprehensive search strategy to capture the products available worldwide. Although this review has its strengths, it is important to acknowledge its limitations. One limitation is that the search was limited to products available in English; thus, it may not be representative of all SMAPs currently available in the global market. Another limitation is that the features identified for each product were limited to resources available on the web; the products were not purchased for testing by our research team. Although this review provides a detailed summary of the features associated with each of the 114 identified SMAPs, it did not evaluate the usability of the products; therefore, it cannot comment on how usable these products are for older adults.

### Conclusions

SMAPs can vary greatly in the features they possess. With an increasing number of SMAPs being introduced into the market, it can be challenging for patients, care partners, and clinicians to determine which is the most appropriate product for medication management. This review can potentially serve as a useful resource for clinicians to become familiar with the essential features of SMAPs, facilitating their ability to recommend a SMAP that aligns with their patients’ specific needs related to medication management.

## Appendix

Multimedia Appendix 1 Databases search strategy.

Multimedia Appendix 2 Google and YouTube search strategy. A detailed list of the features of each product is available on our website.

## Abbreviations

PRISMA: Preferred Reporting Items for Systematic Reviews and Meta-Analyses
SMAP: smart medication dispensing and adherence product
