# Supplementary material for: Key Features of Smart Medication Adherence Products: Updated Scoping Review
Source: JMIR Aging. 2023 Dec 19;6:e50990. doi: 10.2196/50990 (PMC10762620; doi:10.2196/50990)
Supplement: Multimedia Appendix 1 [file aging_v6i1e50990_app1.pdf]

Databases Search Strategy

Databases	Search strategy
PubMed	(medication adherence[mesh] OR medication adherence[tiab] OR medication therapy management[mesh] OR medication therapy management[tiab] OR medication management[tiab]) OR ((medication*[tiab] OR drug*[tiab] OR prescription*[tiab]) AND (adhere*[tiab] OR nonadher*[tiab] OR “non-adher*”[tiab] OR compli*[tiab] OR comply[tiab])) AND (mobile applications[mesh] OR mobile*[tiab] OR smart*[tiab] OR mhealth[tiab] OR technology[mesh] OR technolog*[tiab] OR electronics[mesh] OR electronic*[tiab] OR computers, handheld[mesh]) AND (dispens*[tiab] OR product*[tiab] OR device*[tiab] OR delivery unit[tiab] OR blister*[tiab] OR bottle*[tiab]) AND English[lang] AND 2019:2022[edat]
Embase	1. exp medication compliance/<br>2. exp medication therapy management/"<br>3. ("medication therapy managem "nt" "r "medication managem "nt").ti,ab.<br>4. ((medication* or drug* or prescription*) adj3 (adhere* or nonadher* or "non-adher*" or compli* or comply)).ti,ab.<br>5. 1 or 2 or 3 or 4<br>6. exp mobile application/ or exp mobile health application/ 21582<br>7. (mobile* or smart or mHealth).ti,ab. 1<br>8. exp medical electronics/<br>9. exp medical technology/<br>10. exp personal digital assistant/<br>11. (technolog* or electronic*).ti,ab.<br>12. 6 or 7 or 8 or 9 or 10 or 11<br>13. (dispens* or product* or device* or "delivery unit" or blister* or bottle*).ti,ab.<br>14. 5 and 12 and 13<br>15. limit 16 to english language and y"= “2019- Curr"nt”)
Scopus	(TITLE-ABS "("medication therapy managem""" "medication managem "t")) OR TITLE-ABS ((medication* OR drug* OR prescription*) W/3 (adhere* OR nonadher* " "non-adh" " OR compli* OR comply)) AND TITLE-ABS ((mobile* OR smart* OR mhealth OR technolog* OR electronic*)) AND TITLE-ABS ((dispens* OR product* OR device* " "delivery u" " OR blister* OR bottle*))) AND LANGUAGE (english)

This is a Multimedia Appendix to a full manuscript published in the J Med Internet Res Aging.
For full copyright and citation information see http://dx.doi.org/10.2196/50990
