# Supplementary material for: Key Features of Smart Medication Adherence Products: Updated Scoping Review
Source: JMIR Aging. 2023 Dec 19;6:e50990. doi: 10.2196/50990 (PMC10762620; doi:10.2196/50990)
Supplement: Multimedia Appendix 2 [file aging_v6i1e50990_app2.pdf]

Google and YouTube Search Strategy

Search Engine	Search Strategy
Google	1. (medication OR drug OR pill OR prescription) AND (technology OR technologies OR electronic OR electronics OR smart OR mobile OR mHealth) AND (dispense OR dispenses OR dispenser OR dispensers OR dispensing)<br>2. (medication OR drug OR pill OR prescription) AND (technology OR technologies OR electronic OR electronics OR smart OR mobile OR mHealth) AND (product OR products OR device OR devices)<br>3. (medication OR drug OR pill OR prescription) AND (technology OR technologies OR electronic OR electronics OR smart OR mobile OR mHealth) A "D ("delivery "nit"" OR "delivery u" its")<br>4. (medication OR drug OR pill OR prescription) AND (technology OR technologies OR electronic OR electronics OR smart OR mobile OR mHealth) AND (blister OR blisters OR bottle OR bottles)
YouTube	Search words: Smart medication dispensers, smart medication device, smart dispensing delivery, smart blister pack, smart medication vial

This is a Multimedia Appendix to a full manuscript published in the J Med Internet Res Aging.
For full copyright and citation information see http://dx.doi.org/10.2196/50990
